# OCT angiography for the diagnosis and management of choroidal neovascularization secondary to choroideremia

**DOI:** 10.1016/j.ajoc.2021.101042

**Published:** 2021-02-25

**Authors:** Ratnesh Ranjan, Shishir Verghese, Romit Salian, George J. Manayath, Veerappan R. Saravanan, Venkatapathy Narendran

**Affiliations:** Department of Retina and Vitreous Services, Aravind Eye Hospital and Postgraduate Institute of Ophthalmology, Coimbatore, Tamil Nadu, India

**Keywords:** Choroideremia, Choroidal neovascularization, Anti-vascular endothelial growth factor, Ranibizumab

## Abstract

**Purpose:**

To describe the multimodal imaging findings and treatment outcomes in choroidal neovascularization secondary to Choroideremia.

**Observations:**

A 13-year-old male presented with reduced visual acuity in the left eye. He gave a history of nyctalopia. His best-corrected visual acuity (BCVA) was 20/20 in the right eye and 20/40 in the left eye. Based on multimodal imaging, the diagnosis of choroideremia in both eyes with a subfoveal choroidal neovascularization in the left eye was made. He underwent five intravitreal anti-vascular endothelial growth factor (VEGF) injections of Ranibizumab over a period of 3 years, with the final injection given due to recurrence of neovascularization. Post-treatment, his BCVA improved to 20/20 in the left eye with regression of the neovascular network.

**Conclusions and importance:**

This case highlights the role of OCTA in diagnosis of choroidal neovascularization in choroideremia as well as its successful management with anti-VEGF injections with long term follow up.

## 1 Introduction

Choroideremia, an X-linked chorioretinal dystrophy, is characterized by extensive atrophy of the choriocapillaris, retinal pigment epithelium (RPE) and photoreceptors with relative sparing of the inner retinal layers.^1^^,^^2^ Fundus changes are seen as early fine retinal pigment mottling followed by underlying choroidal atrophy, which start in mid-periphery and slowly progress centrally. Affected males typically present with nyctalopia in the first decade of life, followed by peripheral visual field loss. The central vision usually remains unaffected due to the preservation of macular anatomy till the 5th -7th decade of life.

Loss of central visual acuity at a young age in choroideremia is a rare manifestation of the disease which may occur due to development of cystoid macular oedema or choroidal neovascularization (CNV). We describe the role of multimodal imaging in the diagnosis and monitoring of a case presenting with CNV secondary to choroideremia, and the long-term outcomes following prompt treatment with anti-vascular endothelial growth factor (VEGF) injections.

## 2 Case report

**Fig. 1:**
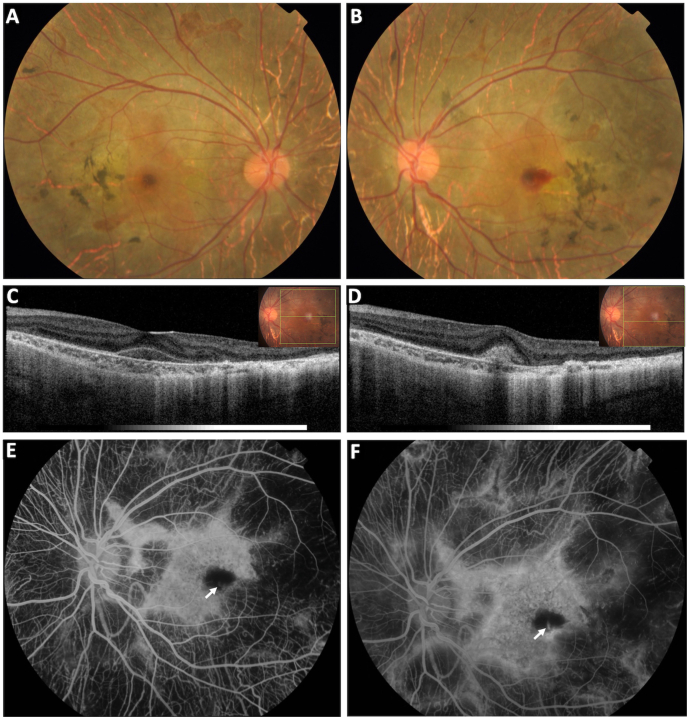
Colour fundus photograph of both eyes showing diffuse chorioretinal atrophy sparing an island of perifoveal tissue in both eyes with small subretinal hemorrhage adjacent to the fovea in the left eye [A, B]. Swept source ocular coherence tomography (SS-OCT) of the LE showing subretinal hemorrhage [C]. SS-OCT through an inferior section showing the type 2 choroidal neovascularization (CNV) complex along with temporal choriocapillaris thinning and loss of RPE and the photoreceptor layer sparing the perifoveal area [D]. Fundus fluorescein angiogram (FFA) of the LE; early phase showing large peripheral areas of missing choriocapillaris appearing hypofluorescent next to bright central island of perfused choriocapillaris appearing hyperfluorescent and a small abnormal neovascularization (arrow) at the edge of foveal avascular zone with mild late leakage suggestive of classic CNV. [E, F]. (For interpretation of the references to colour in this figure legend, the reader is referred to the Web version of this article.)

**Fig. 2:**
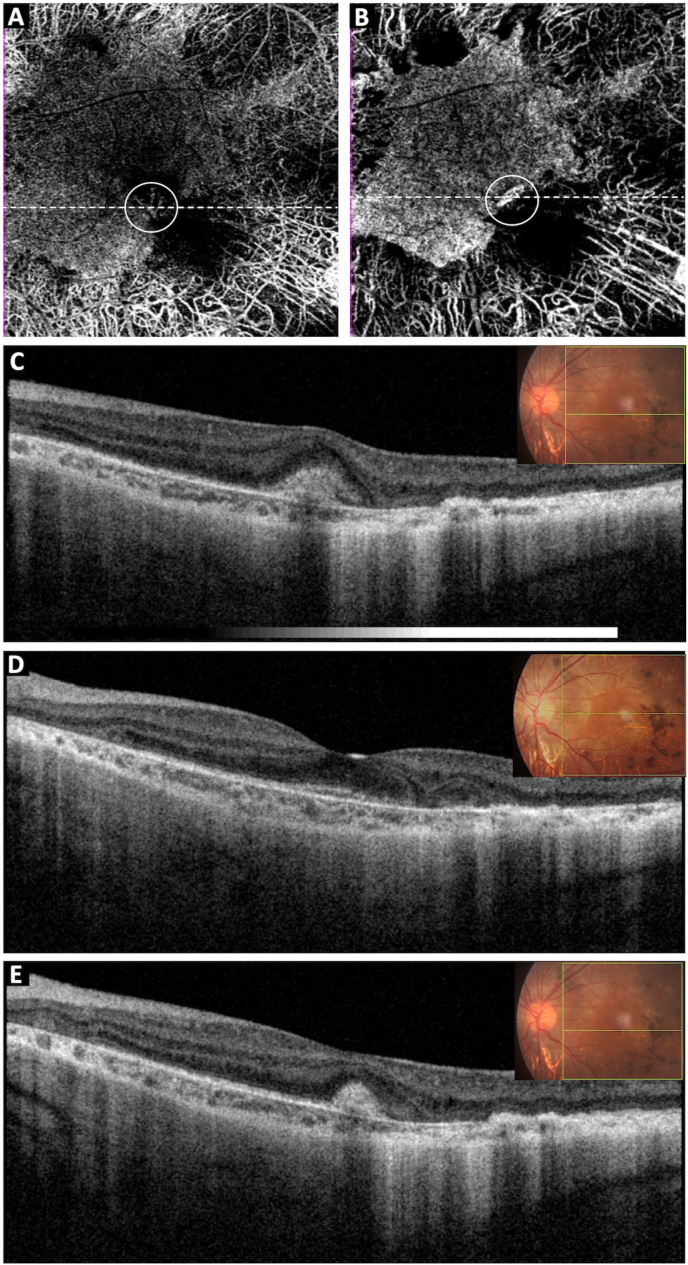
Ocular coherence tomography angiography (OCTA) of the left eye at presentation showing a neovascular network at the level of choriocapillaris, adjacent medium to large choroidal vessels are easily visible due to choriocapillaris attenuation [A]. OCTA, following 4 injections of Ranibizumab, showing well shrunken small vascular network at the level of choriocapillaris [B]. Corresponding pre-treatment ocular coherence tomography showing the CNV complex corresponding to OCTA enface image (white line) [C]. Post-treatment OCT horizontal section showing resolution of subretinal hemorrhage in the sufoveal area [D]; and reduction in size of the CNV complex corresponding to the OCTA enface image (white line) [E].

**Fig. 3:**
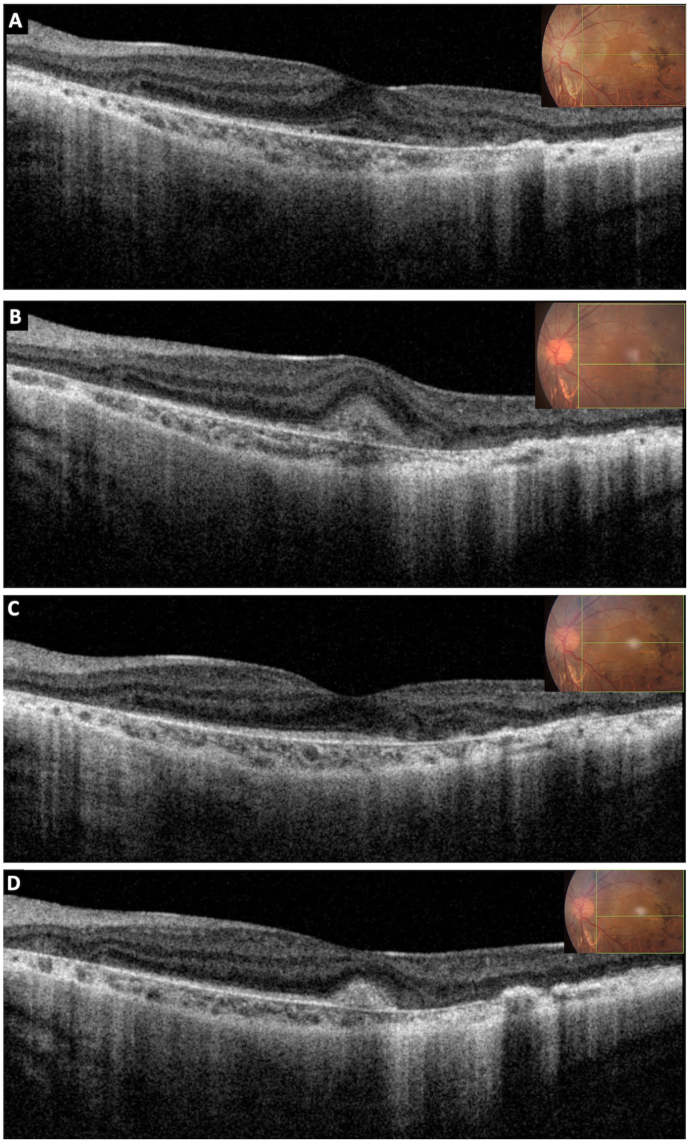
Ocular coherence tomography (OCT) of the left eye, 6 months later, showing disease reactivation seen as the presence of subretinal fluid and increase in size of the CNV complex [A, B]. OCT, at 6 months after repeat injection, showing stabilization of the disease with a small hyperreflective lesion consistent with fibrosis [C, D].

A 13-year-old boy, presented with complaints of reduced vision in the left eye (LE) for one month. He gave a history of night blindness for the past one year. He did not have any systemic illnesses. Both the parents and his only sibling were asymptomatic and were normal on ocular examination. On examination, his best-corrected visual acuity was 20/20 in the right eye (RE), and 20/40 in the LE. Anterior segment examination was unremarkable in both eyes. Fundus examination of both eyes revealed pigment clumping with areas of widespread chorioretinal atrophy with visible network of large choroidal vessels sparing a large island of perifoveal area with pseudopodia like extensions suggestive of choroideremia. [Fig. 1A and B]. Additionally, a small subretinal hemorrhage was seen juxtafoveally in the LE [Fig. 1B]. A full field electroretinogram was done initially which revealed a reduced scotoptic response. Swept source ocular coherence tomography (SS-OCT) showed an overall retinal and choroidal thinning with loss of photoreceptor layers sparing the macula in both eyes. SS-OCT of the LE revealed the presence of a type 2 CNV complex subfoveally with small subretinal hemorrhage and subtle subretinal fluid [Fig. 1C and D]. Fluorescein angiogram revealed scalloped areas of missing choriocapillaris in the mid periphery appearing hypofluorescent next to a bright hyperfluorescent central island of perfused choriocapillaris in both eyes. A faint lacy pattern of vessels at the edge of foveal avascular zone with late leakage confirmed the presence of subfoveal CNV in the LE [Fig. 1E and F]. OCT angiography images were procured using the Zeiss Cirrus HD-OCT 5000 (Zeiss Meditec, Dublin, CA) with Angioplex using 6 × 6 sections at the level of the RPE-RPE fit which also revealed a neovascular network at the level of the outer retina in the corresponding location in the LE [Fig. 2A] along with widespread choriocapillaris attenuation and visualization of the medium and large choroidal vessels with relative macular sparing in both eyes. With a diagnosis of active CNV secondary to choroideremia in the LE, the patient was started treatment with intravitreal anti-VEGF injection (0.05 ml of Ranibizumab) in the same eye after taking the written consent from the legal guardian. CNV showed regression with complete disappearance of subretinal fluid, subretinal hemorrhage and reduction in the size of vascularity of the lesion, which required 4 injections of Ranibizumab at one-monthly interval [Fig. 2 B, C, D]. At this stage, his BCVA in the LE improved to 20/20 with OCT angiography confirming the shrinkage of neovascular complex [Fig. 2B]. A reactivation of CNV was noted with reappearance of subretinal fluid at month six after the 4th injection leading to reduction in BCVA to 20/30 in the LE [Fig. 3A and B]. A repeat intravitreal injection of Ranibizumab resulted in resolution of subretinal fluid and improvement of BCVA to 20/20 in the LE. Since then, patient has maintained his BCVA (20/20) in the LE without any recurrence of CNV activity during a follow up period of 30 months [Fig. 3 C, D].

## 3 Discussion

CNV is a rare manifestation of choroideremia with only a limited number of case reports available in the literature.^3^ The pathogenesis of CNV is presumed to be secondary to RPE degeneration, the extent of which is greater than the extent of choriocapillaris loss towards macula.^4^ Recently, similar findings have been described in the non-invasive OCT angiography-based assessment of atrophic changes in choroideremia. A disruption in the RPE-Bruch's membrane complex can give rise to CNV from the underlying intact choriocapillaris,^5^ as seen in our case where angiographically the CNV arises from the junction of intact and atrophic RPE. Although uncertain, RPE, macrophages, Muller cells, endothelial cells and fibroblasts are suspected as the potential source of VEGF production leading to growth of neovascular tissue.^6^^,^^7^ With the RPE being intact at the fovea, Patel et al. suggested fovea and perifoveal area as the main site of VEGF production.^5^ This corroborates with the findings in other reports where choroideremia-associated CNVs were subfoveal in location, similar to our case.

Choroideremia-related CNV may present with a small subretinal hemorrhage and a subretinal neovascular membrane, as seen in our patient. Presentation with significant exudation in the form of clinical and tomographic evidence of subretinal and intraretinal fluid is rare. Fluorescein angiography also reveals only minimal neovascular leakage due to the atrophic nature of surrounding choriocapillaris.^8^, ^9^, ^10^ The presence of subtle subretinal fluid on OCT and minimal leakage from a small CNV complex on fluorescein angiography in this case were in accordance with the previous reports.^8^, ^9^, ^10^ In recent years, OCT angiography is successfully being used for diagnosing and monitoring treatment of CNVs secondary to hereditary chorioretinal disease. Till now, only Patel et al. have reported use of OCT angiography in the management of CNV in choroideremia.^5^ Pre-treatment OCT angiography of our case showed a neovascular network at the level of the RPE, and the attenuated choriocapillaris sparing the macula. Since CNVs secondary to choroideremia can often go unnoticed due to their small size and limited exudation, a careful approach with multimodal imaging would be helpful in early diagnosis of these lesions.

With prompt anti-VEGF treatment with Ranibizumab, our patient has maintained a visual acuity of 20/20 in the affected eye. Owing to propensity for spontaneous resolution, CNVs secondary to choroideremia have been just observed in older reports.^4^^,^^8^, ^9^, ^10^ However, with introduction of anti-VEGF agents, these lesions may be managed more effectively. In literature, there are only two case reports describing the use of anti-VEGF for treatment of choroideremia-related CNV. Palweja et al. reported a case of a 13-year-old boy with choroideremia and subfoveal CNV in the LE with a visual acuity of 20/150 which remained unchanged despite 12 injections of Bevazizumab.^3^ Patel et al. also described a 13-year-old boy with choroideremia and type 1 CNV in the LE. They described the OCTA pattern to be a linear neovascular network which required 13 injections of Bevazizumab with visual acuity improving from 20/200 to 20/100.^5^ Both these cases had limited improvement in visual acuity despite multiple anti-VEGF injections for CNV stabilization.^3^^,^^5^ Unlike the previous reports, our patient needed only 5 injections over a period of 3 years for the stabilization of CNV regression and the maintenance of very good visual acuity. This might be attributed to the initiation of prompt treatment with anti-VEGF at the early stage. The reduction in the size of neovascular network was confirmed on OCTA and OCT showing a small juxtafoveal subretinal hyper-reflective lesion consistent with CNV regression following therapy.

To conclude, the preservation of the central visual acuity is of prime importance in an eye with extensive peripheral visual field loss due to choroideremia. Multimodal imaging especially OCTA is useful for close monitoring in choroideremia, which can help in early diagnosis of CNV, a rare manifestation of the disease. Initiating prompt treatment with anti-VEGF would help to maintain good central acuity by stabilizing the CNV complex and preventing subretinal fibrosis.

## Patient consent

An informed consent has been obtained from the legal guardian for publication of data and ocular images.

## Funding

No funding or grant support

## Financial disclosures

All the authors of this case report have no financial disclosures (R.Ranjan, S.Verghese, R. Salian, G.J Manayath, V.R Saravanan, V.Narendran).

## Authorship

All authors attest that they meet the current ICMJE criteria for authorship.
